# Meta-atom loaded circularly polarized triple band patch antenna for Wi-Fi, ISM and X-band communications

**DOI:** 10.1016/j.heliyon.2024.e28906

**Published:** 2024-03-28

**Authors:** Suresh Angadi, Karteek Viswanadha, Ravikumar Chinthaginjala, Dhanamjayulu C, Kim Tai-hoon, Kumar S

**Affiliations:** a Department of ECE, Delhi Technological University, New Delhi, India; b School of Electronics Engineering, Vellore Institute of Technology, Vellore, 632014, India; c School of Electrical Engineering, Vellore Institute of Technology, Vellore, 632014, India; d School of Electrical and Computer Engineering, Yeosu Campus, Chonnam National University, 50 Daehak-ro, Yeosu-si, Jeollanam-do, 59626, Republic of Korea; e Data Science Research Laboratory, BlueCrest University, Monrovia, Liberia, 1000

**Keywords:** Meta-atom, Stub, Dumbbell, Circular polarization, Wi-Fi

## Abstract

Microstrip antennas usually suffer from high losses, gain, and efficiency degradation. It is a challenging task to miniaturize the patch antenna without degrading the performance parameters. To mitigate the above problems, a microstrip patch antenna loaded with stubs and printed on the ground plane loaded with dumbbell meta-atom is presented in this paper. The proposed double dumbbell meta-atom consists of two complementary split ring resonator (CSRR) cells loaded with rectangular rings. This exhibits the Double Negative (DNG) characteristic at 2.45 GHz. The devised meta-atom possesses dimensions of 0.05λ x 0.03λ at lower giga-hertz range. The meta-atom is further analyzed in CST-Microwave Studio and the corresponding S-parameters are extracted in MATLAB using the Nicolson Ross Weir (NRW) method. The electrical model of the meta-atom is also analyzed using Agilent ADS simulator. Further, two models of the proposed antenna with FR-4 and RT/Duroid-5880 are designed and compared. The proposed patch antenna resonates at three different frequency bands i.e. 2.445 GHz with a 3-dB bandwidth of 110 MHz (2.4 GHz–2.51 GHz), at 5.85 GHz with a 3-dB bandwidth of 730 MHz (5.13 GHz–5.86 GHz), and at 8.83 GHz with a 3-dB bandwidth of 1.83 GHz (7.7 GHz–9.53 GHz). This exhibit peak gains of 2.75dBi, 3.53dBc and 4.36dBi with low cross polarization levels at the said frequencies of operation. Further, the antenna possesses circular polarization in the frequency band (5.15 GHz–5.63 GHz). This antenna is used for Wi-Fi, ISM and X-band communications. The designed prototype is fabricated and tested and bears resemblance to the simulated results.

## 1 Introduction

Microstrip antennas are pivotal devices in the back end of a transmitter. Usually, these antennas are small and occupy less space. These are further miniaturized using different techniques like loading slots, shorting pins, and fractal geometries to make them resonate at multiple frequencies [1]. Though miniaturization introduces multiband features, it has its own disadvantages. Firstly, it degrades the antenna's performance parameters. Secondly, miniaturization depends on the type of substrate used. Nowadays, lossy substrate like FR-4 is widely used to achieve antenna miniaturization due to its cost effectiveness, electrical stability, and reasonable impedance matching [2]. Though FR-4 achieves reasonable degree of miniaturization, it deteriorates overall output of the antennas. Further, FR-4 is usually used up to 2 GHz to achieve best results. Beyond 2 GHz, FR-4 exhibits radiation leakages leading to gain degradation and distortion of radiation patterns. Further, a detailed literature review on pre-existing FR-4 based patch antennas is presented in the next section. To mitigate the problems, many pre-existing structures are proposed. The proposed structures consist of meta-surfaces embedded inside lossy FR-4 substrate leading to superstrate. Though FR-4 based superstrates act as a respite to the lossy conventional FR-4 based patch antennas, the dielectric losses and radiation leakages beyond 2 GHz are inevitable. To achieve miniaturization along with the enhanced performance parameters, an appropriate substrate with low loss and a high-frequency range must be used. RT/Duroid-5880 acts a suitable candidate to overcome the above problems. At the same time, RT/Duroid-5880 based conventional antenna cannot achieve the desired results. To achieve the desired results, a dumbbell meta-atom is loaded on the ground plane.

This literature discusses different types of FR-4 based single-band, dual-band and tri-band metamaterial patch antennas resonating at 2.45 GHz [[3], [4], [5], [6], [7], [8], [9], [10], [11], [12], [13], [14], [15], [16], [17], [18], [19], [20], [21], [22], [23], [24], [25], [26], [27]].

Different types of patches loaded with CSRR metamaterial are proposed in Refs. [[3], [4], [5]]. These antennas are designed on FR-4 substrate having high di-electric constant. High gain and efficiency are usually achieved by increasing overall radiating area of the antenna structure. This is undesirable when the objective is to miniaturize a patch antenna. Further, the implementation of FR-4 in these structures increases the electrical length of these antennas, thereby deteriorating the performance parameters. The antenna proposed in Ref. [6] acts as a suitable solution to the problems faced by conventional FR-4 based patch antennas. A FR-4 based complementary split ring resonator (CSRR) and complementary closed ring resonator (CCRR) metamaterial inspired tri-band patch antenna in Ref. [6] achieves the desired gain enhancement. Despite achieving the gain enhancement, the antenna suffers from gain instability over the resonant bands. Structures proposed in Refs. [[7], [8], [9], [10], [11]] possess stable gains and a reasonable degree of compactness. But these antennas have high magnitude of cross polarization levels which highly undesirable. In Ref. [12], FR-4 based CPW fed tri-band meander patch antenna is proposed. The antenna is printed on substrate with total dimensions of 23 mm × 23 mm. The antenna possesses reasonable frequency ratio. Apart from CPW fed antennas, metamaterial inspired antenna are deployed to fulfill the modern-day wireless requirements. Metamaterial embedded antenna in Ref. [13] with overall area of 40 mm × 30 mm resonates at 2.46 GHz. This structure possesses a peak gain of 3.3dBi. Antenna in Ref. [14] provides a good insight on the effect of the number of metamaterial cells on latter's performance. The antenna in Ref. [14] resonates at single frequency i.e. 2.45 GHz when two CSRR cells are loaded. The antenna further resonates at 2.45 GHz and 2.72 GHz when it is loaded with a single CSRR cell. With the above design, a peak gain of 2.33dBi is achieved with unstable radiation patterns and efficiency degradation. It is quite often challenging to achieve stable radiation patterns in different bands. Tri-band patch antenna in Ref. [15] achieves stable radiation patterns in the frequency bands of 2.5 GHz–2.7 GHz, 3.3 GHz–3.8 GHz, and 5.3 GHz–5.9 GHz. This structure is designed using a composite right left-handed (CLRH) transmission line (TL). This achieves gains ranging from 0.7dBi to 1.9dBi in the said frequency bands. Constant gains over a range of frequencies lead to stable efficiencies. At the same time, constant efficiencies do not assure low radiation leakages. Therefore, it is utmost desirable to achieve high radiation efficiencies with low radiation leakages. Metamaterial inspired patch antennas [[16], [17], [18], [19], [20]] achieve stable and high efficiencies of over 90% along with stable radiation patterns. Metamaterials are directly deployed to radiate on their own without the support of patch structure. In some conventional patch antennas, metamaterials are modified to act as ground plane. One such design is proposed in Ref. [21] where a split ring resonator connected to a strip line is deployed as ground plane. This antenna resonates at 2.51 GHz and 3.41 GHz and possesses peak gains of 1.18dBi and 2.2dBi. Unfortunately, this design is prone to huge efficiency variations at the frequencies of operation.

Different shapes of metamaterials are amalgamated to achieve required degree of compactness with minimal fluctuation in gain and efficiency. Metamaterials are often used as impedance converter to achieve compactness, omnidirectional feature, and reasonable gains. Two such designs are proposed in Refs. [22,23]. In Ref. [22], circular SRR is embedded inside rectangular SRR to achieve dual-band feature. This resonates at 2.47 GHz, and 3.62 GHz with peak gains of 2.25dBi and 0.88dBi respectively. In Ref. [23], an impedance converter-based antenna is proposed. In this design, a Y shaped radiator is embedded inside a high impedance broken circular ring. This exhibits resonance at 2.4 GHz, 3.5 GHz, and 4.9 GHz with peak gains of 3.6dBi, 2.95dBi, and 3.96dBi respectively.

The antennas in Ref. [24] achieve high degree of compactness at 2.45 GHz. The structure consists of a triangular patch loaded with SRR. But this antenna is prone to gain degradation. This problem is addressed by antennas in Refs. [25,26]. Different types of metamaterials like CSRR, and complementary double negative metamaterial are used in antennas proposed in Refs. [25,26]. These antennas act as a tradeoff between the compactness and gain. Antenna in Ref. [27] provides a reasonable tradeoff between compactness and gain. Rectangular patch antenna in Ref. [27] is designed to have a wide area of 4340 mm^2^ and is designed to resonate at 2.45 GHz with a peak gain of 3.12dBi.

The proposed patch antenna acts as a suitable candidate to overcome the problems faced by the antennas discussed in the literature. The offered antenna is loaded with dumbbell meta-atom to achieve high degree of compactness and improved performance parameters. The proposed meta-atom shows negative permeability and permittivity at 2.45 GHz. Overall volume of the structure is 12 mm × 15 mm x 1.524 mm. The antenna structure resonates at 2.445 GHz, 5.85 GHz, and 8.83 GHz with the peak gains of 2.75dBi, 3.53dBc and 4.36dBi at the said frequencies of operation. Further, the antenna possesses circular polarization in the frequency band (5.15 GHz–5.63 GHz). This antenna is used for Wi-Fi, ISM and X-band communications.

## 2 Design of the proposed meta-atom loaded patch antenna

**Fig. 1:**
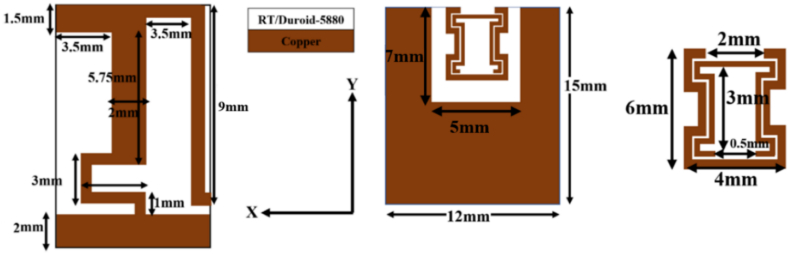
Top and bottom views of proposed prototype.

The developed protype comprises of a meander shaped radiator loaded with a quarter wavelength ‘L’ shaped stub line. The structure is designed using RT/Duroid-5880 (ε_r_ = 2.2) with a loss tangent of 0.0004. The proposed prototype is loaded on the ground layer with dimensions (L_G_ x W_G_ x w) of 12 mm × 15 mm x 0.035 mm. A simple half-wavelength meander shaped patch is designed using a conventional method. The meander patch antenna is designed to have a length of 10 mm and resonate at 8.8 GHz. A portion with dimensions of 5 mm × 3 mm is cut beneath the patch and at upper end of the ground layer as shown in Fig. 1. A meta-atom is placed inside the slot to achieve the miniaturization and tri-band feature of the proposed patch antenna. A brief study of proposed meta-atom is carried out in Ref. [28]. Where, the impact of the dumbbell meta-atom on the performance of a stub loaded patch antenna is briefly studied. The position of meta-atom determines the degree of miniaturization and improves the performance parameters of the proposed antenna structure. The effect of position of the meta-atom on miniaturization is studied in the parametric analysis section. The patch antenna possesses an input impedance of 138 Ω. The impedance matching between the SMA connector and the patch antenna is achieved with the help of a quarter wavelength feed line of dimensions 12 mm × 2 mm and having an impedance of 83.36 Ω is connected to the patch antenna. An 'L'-shaped radiator stub (resonator) is connected to the meander patch antenna adds resonant frequency at 5.8 GHz. The dimensions of resonator are obtained from Ref. [29]. The length of the resonator is 9 mm. Further, a dumbbell meta-atom is placed on the ground to introduce resonant frequency resonate at 2.45 GHz. A detailed discussion on proposed meta-atom is presented in the subsequent sections. The resonant frequencies corresponding to the meander section, L-stub line section and meta-atom are computed using the equations presented in Ref. [29].

## 3 Meta-atom design

The design methodology of the proposed meta-atom is based on the metamaterial proposed in Ref. [30]. The proposed dumbbell meta-atom was loaded on the ground plane as shown in Fig. 1. The ground plane is printed below RT/Duroid-5880 substrate. The proposed meta-atom comprises of two complementary split ring resonators (CSRR) connected via two concentric rectangular strips of length 3 mm. RT/Duroid-5880 plays an important role in achieving impedance matching, electric signal stability and low dielectric loss. The proposed meta-atom is formed by connecting two CSRR cells of dimensions 4 mm × 2 mm.Each CSRR cell has a split gap of 0.5 mm. The split gap was based on iterations to achieve optimization that leads to resonance at the desired frequency.

## 4 a. Validation of the meta-atom

The following formulae are used to obtain the propagation properties of the proposed meta-atom.

(1) $$\text{Reflection}\;\text{coefficient}\;\left(\rho \right)=\frac{Z-1}{Z+1}$$

where Z is the normalized impedance.

(2) $$Z=\sqrt{\frac{\mu_{r}}{\varepsilon_{r}}}$$

Return loss (s_11_) is computed by:

(3) $$S_{11}=\frac{\left(1-\rho^{2}\right)Z}{1-\rho^{2}Z}$$

Transmission co-efficient (s_21_) is given by

(4) $$S_{21}=\frac{\left(1-Z^{2}\right)\rho }{1-Z^{2}\rho }$$

(5) $$V_{1}=S_{11\;}+S_{21}$$

(6) $$V_{2}=-S_{11\;}+S_{21}$$

where V_1_ and V_2_ are sum and differences of reflection co-efficient and insertion loss.

(7) $$\varepsilon_{r\;}=\frac{C}{j\pi fd_{m}}\times \left[\frac{1-V_{1}}{1+V_{1}}\right]$$

(8) $$\mu_{r\;}=\frac{C}{j\pi fd_{m}}\times \left[\frac{1-V_{2}}{1+V_{2}}\right]$$

(9) $$f_{r}=\frac{1}{2\pi \sqrt{LC}}$$

(10) $$L\left(\text{nH}\right)=2\times 10^{-4}\;l_{m}\;[1.193+\log\;\_\left(e\right)\;\left[\frac{l_{m}}{w_{m}+t_{m}}\right]+0.02235\left[\frac{w_{m}+t_{m}}{l_{m}}\right]\times K_{g}$$

where K_g_ is the adjustment factor and can be found by this equation K_g_ = 0.57–0.145ln $\frac{w_{s}}{t_{s}}$ where w_s_ and t_s_ are the width and thickness of the substrate.

(11) $$C=\frac{\varepsilon_{o}\varepsilon_{r}A}{d_{m}}$$

where ‘dm’ is the height of the meta-atom, ‘fr’ is the resonant frequency and ‘c’ is the velocity of light. The metal strips on the meta-atom corresponds to the inductance and the strip gap corresponds to the capacitance. lm, wm and tm are the length, width, and thickness of the strip.

With the help of equations (1), (10), (11), (2), (3), (4), (5), (6), (7), (8), (9), resonant frequency of the proposed meta-atom can be validated.

(12) $$\text{The}\;\text{resonant}\;\text{frequency}\;\left(f_{1}\right)\;\text{corresponding}\;\text{to}\;\text{meta}-\text{atom}=\frac{1}{2\pi \sqrt{\text{LC}}}$$

The computed frequency is obtained as 2.48 GHz which is in proximity with simulated and measured results.

Further, the impact of the meta-atom on the performance of the proposed antenna structure is studied through the developmental stages.

**Fig. 2:**
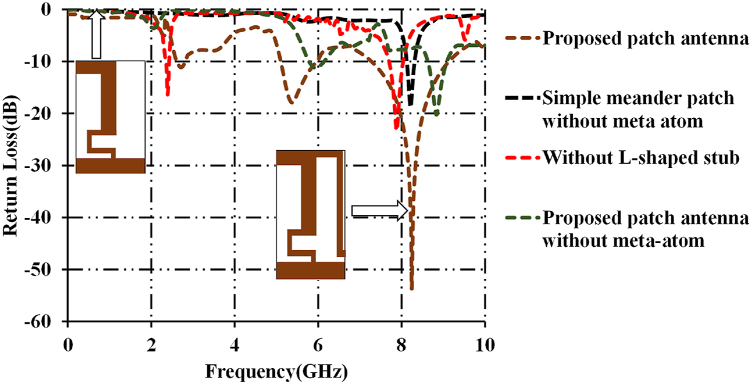
Evolution stages with their return losses.

The variation in the impedance bandwidths of progressively developed designs of the proposed prototype is shown in Fig. 2. A simple meander shaped patch antenna with meta-atom resonates at 8.8 GHz as shown in the black dotted curve. When the simple meander patch antenna is loaded with the dumbbell meta-atom, the antenna resonates at 2.45 GHz and 7.95 GHz as shown in the red dotted curve. When the antenna is connected to a quarter wavelength ‘L’-shaped resonator with the conventional ground plane, the antenna resonates at 5.89 GHz and 8.91 GHz as shown in the green dotted curve. The proposed patch antenna resonates at 2.44 GHz, 5.79 GHz and 8.8 GHz as shown in the brown dotted curve. The proposed antenna possesses impedance bandwidths of 110 MHz, 1.12 GHz, and 2.3 GHz in the frequency ranges of 2.4GHz–2.5 GHz, 4.87 GHz–6 GHz, and 7.2 GHz–9.5 GHz respectively.

The basic design of the proposed patch antenna is inspired from the antenna designed in Ref. [29]. Where, the antenna possesses a total volume of 198.12 mm^3^ and resonates at 6.8 GHz, 9.51 GHz ad 9.89 GHz. Whereas, the proposed design has total volume of 180 mm^2^. The idea behind the proposed patch antenna is to optmize the design and to shift the resonating frequency from 6.8 GHz to 2.45 GHz. The design in Ref. [28] is modified further using meta-atom to achieve desired improvisation.

**Fig. 3:**
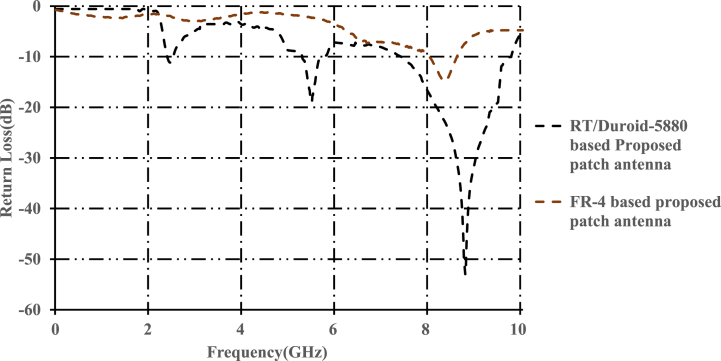
Return loss of FR-4 and RT/Duroid-5800 based proposed patch antenna.

Fig. 3 shows the impedance bandwidths of FR-4 based and RT/Duroid-5880 patch antenna. The FR-4 based patch antenna resonates at 8.45 GHz with an impedance bandwidth of 224 MHz (8.21 GHz–8.53 GHz). As discussed earlier, FR-4 based patch antenna is not suitable for the applications beyond 2 GHz. On the other hand, RT/Duroid-5880 based patch antenna is successful in achieving the desired multiband feature. Another observation is that the FR-4 based antenna achieved low impedance bandwidth when compared to that of the proposed patch antenna. Plots of gains and efficiencies of FR-4 based and RT/Duroid-5880 based patch antenna antennas are discussed in the further sections.

**Fig. 4:**
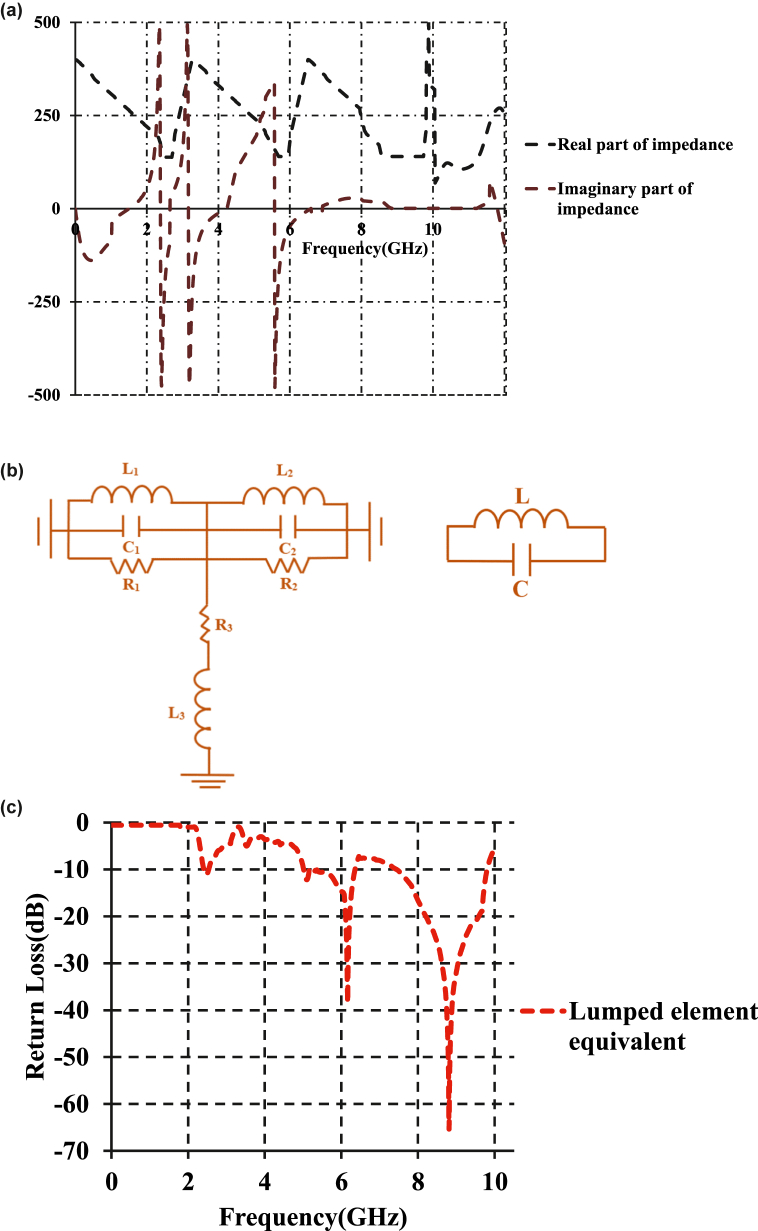
(a)Real and imaginary impedances of the proposed patch antenna Fig. 4(b).L-C equivalent of proposed antenna structure (left) and L-C equivalent circuit of a dumbbell meta-atom (right).(R_1_ = 83.5Ω, L_1_ = 1.5 nH, C_1_ = 0.5 pF, R_2_ = 100Ω, L_2_ = 0.5 nH, C_2_ = 10 pF, R_3_ = 5Ω, L_3_ = 0.1 nH) Fig.4(c). Return loss of the equivalent electrical model.

Fig. 4 a shows the impedance plots of the proposed patch antenna. It is evident at the imaginary impedance is zero at the frequencies of resonance i.e. at 2.45 GHz, 5.78 GHz, and 8.82 GHz. The lumped element model is extracted using the method proposed in Ref. [31]. Impedance plots act as a basis for the derivation of electrical model of the proposed patch antenna. The electrical model of the designed antenna prototype is shown in Fig. 4 b. The equivalent circuit is designed by obtaining the lumped element values from the real and imaginary impedance plots of designed antenna in CST-MW Studio. The model is implemented in Agilent ADS-2016 version in the schematic platform. Two L-C tanks represent meander patch, meta-atom, and the ‘L’-shaped stub line. The inductance, capacitance, and resistance (L_1_, C_1_, R_1_) correspond to the meander patch portion. The lumped elements (R_2_, L_2_, C_2_) correspond to the ‘L’-shaped resonator section and inductance and resistance (L_3_, R_3_) correspond to the feed line section attached to the meander patch. L_3_ and R_3_ also contribute to the resonance. Tuning the lumped elements [(L_1_, C_1_, R_1_) (R_2_, L_2_, C_2_) (L_3_, R_3_)] corresponding to the radiator portion effects the dimensions of latter. The change in the dimensions shifts the resonant frequency. Change in the value of any of the lumped element disturbs the impedance matching and increases the return loss.

Fig. 4 c shows the impedance bandwidth curve of the equivalent electrical model of the proposed prototype. This possesses resonance at 2.4 GHz, 6.02 GHz, and 8.92 GHz. Impedance bandwidths of 110 MHz, 820 MHz and 1.5 GHz are obtained between 2.35GHz and 2.45 GHz, 5.2GHz–6.02 GHz, and 7.4GHz–9.7 GHz. The electrical equivalent is further optimized using the ADS schematic tuner.

## 5 Parametric analysis

**Fig. 5:**
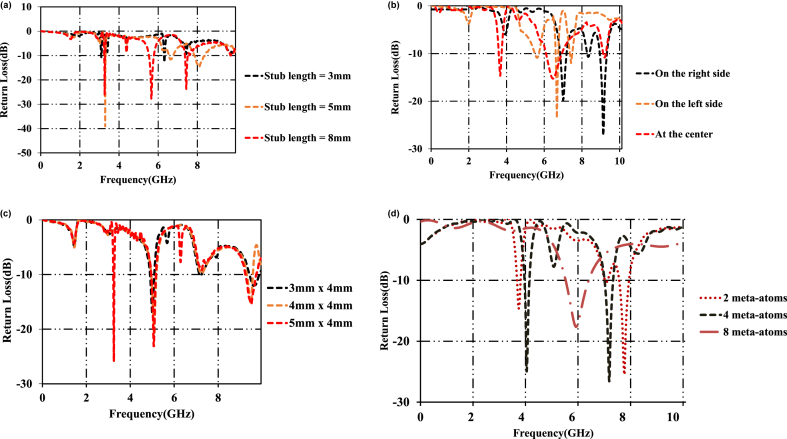
(a)Influence of the stub length on impedance bandwidth. Fig. 5(b). Influence of the position of the meta-atom on the impedance bandwidth. Fig. 5(c). Influence of the overall area of the meta-atom on the impedance bandwidth. Fig. 5(d). Influence of number of meta-atoms on the impedance bandwidth.

The multiband feature in the developed design depends on the length of the stub, the position, and dimensions of the unit cell meta-atom. The impact of the above design parameters on the impedance bandwidth is studied. The variation in return loss with the variation in the design parameters is observed through CST-Microwave studio. Fig. 5(a) presents the influence of the length of the 'L'-shaped resonator on the impedance bandwidth. The stub length is varied between 3 mm and 8 mm. The first resonant frequency i.e. 2.45 GHz is not affected much. There is a shift in the second and third-second frequencies. When the stub length is tuned to 3 mm, the second resonant frequency is relocated to 6.23 GHz and the third resonant frequency is deviated beyond 10 GHz. When the stub length is 5 mm, second and third frequencies are shifted to 6.34 GHz and 8.1 GHz. Similarly, the third frequency is shifted to 7.76 GHz with a stub length of 8 mm.

The influence of the position of the meta-atom on the impedance bandwidth is shown in Fig. 5(b). The position of the metamaterial highly affects the resonant frequencies of the meander section. Meta-atom loaded in the upper quadrant (right side) of the ground layer shifts the frequencies to 7.1 GHz, 8.21 GHz, and 9.52 GHz. A miniaturization of 6.6% is achieved with this design. When the meta-atom is shifted towards left side, the antenna structure radiates at 5.8 GHz, 6.72 GHz and 7.74 GHz. With this design, a miniaturization of 14.94% is achieved. Meta-atom placed at the centre shifts the resonant frequencies to 3.82 GHz, 6.34 GHz and 9.05 GHz. A miniaturization of 74% is achieved in this analysis. To achieve the desired optimization, the position of the meta-atom is displaced from the centre vertically. Proposed patch antenna achieves a miniaturization of 177% when the meta-atom is placed at the bottom.

Fig. 5(c) Shows the change in the return with the variation in the overall area of the meta-atom. The shift in the operating frequencies is observed with the variation in the dimensions of the meta-atom. The length of the meta-atom is varied from 3 mm to 5 mm. By tuning the overall length of the meta-atom to 3 mm, the proposed prototype is resonant at 5.2 GHz, 7.21 GHz, and 9.86 GHz. By tuning the overall length of the meta-atom to 4 mm, the propose radiating patch is resonant at 5.26 GHz and 9.86 GHz. By tuning the overall length of the meta-atom to 5 mm, the antenna is resonant at 3.46 GHz, 5.32 GHz, and 9.91 GHz. Hence, the dimensions of the meta-atom are optimized to 6 mm × 4 mm.

Fig. 5(d) shows the impact of number of meta-atoms on the return loss of the proposed antenna. The study is carried out for 2-,4- and 8-meta-atoms. Increasing the number of atoms diminishes the multi-band feature. Increasing the number of meta-atoms beyond 8- yields unacceptable results. Hence, those results are not reported in this paper.

## 6 Outcomes and disquisitions

**Fig. 6:**
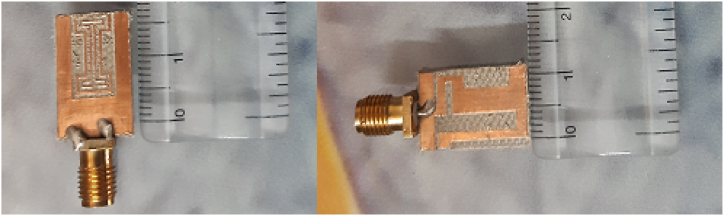
Fabricated prototype.

The proposed prototype is devised using the CST-MWS 2016 version and fabricated with the dimensions obtained after requisite parametric analysis and necessary optimization. Fig. 6 presents the fabricated prototype.

**Fig. 7:**
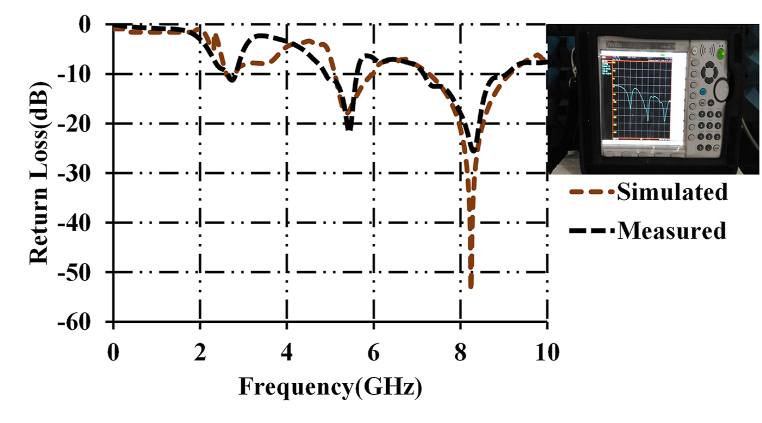
Return losses of the designed antenna.

Fig. 7 presents the results of impedance bandwidths of the developed prototype. The measured and simulated results are in proximity. The minor variations are due to structural discontinuities, the quality of the feed and any fabrication inaccuracies, and the minuscule error in the measured results particularly at the third resonant frequency (8.8 GHz) is observed because of disruption in the fabrication process, which includes systematic errors in SMA connector. The operational bandwidths of the developed prototype are 110 MHz, 730 MHz, and 1.83 MHz in the frequency bands of (2.4 GHz–2.51 GHz), (5.13 GHz–5.86 GHz), and (7.7 GHz–9.53 GHz) respectively. Whereas, the simulated bandwidths of the developed prototype are 112 MHz, 738 MHz, and 1.82 GHz in the frequency bands of (2.42 GHz–2.54 GHz), (5.14 GHz–5.87 GHz), and (7.68 GHz–9.5 GHz) respectively. The measured and simulated results are in proximity. The behavior of the designed antenna is further analyzed using surface current distributions.

**Fig. 8:**
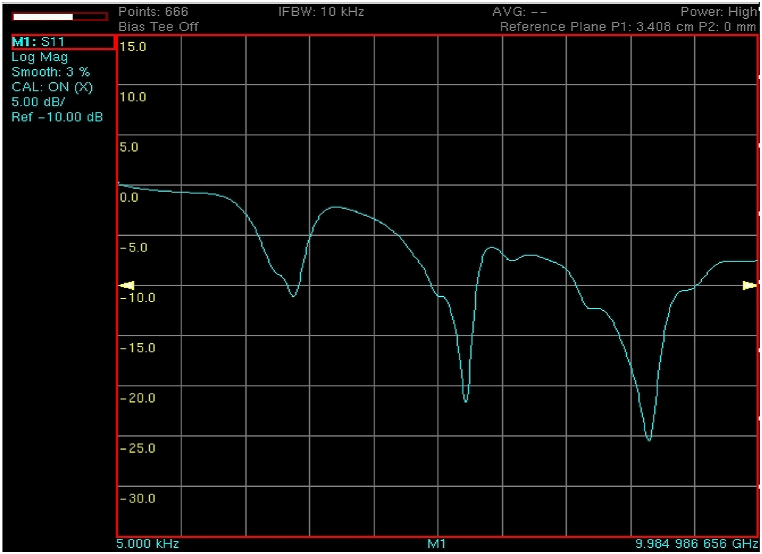
The measured result of the fabricated prototype.

The snapshot of the measured result of the return loss of the fabricated prototype is shown in Fig. 8.

**Fig. 9:**
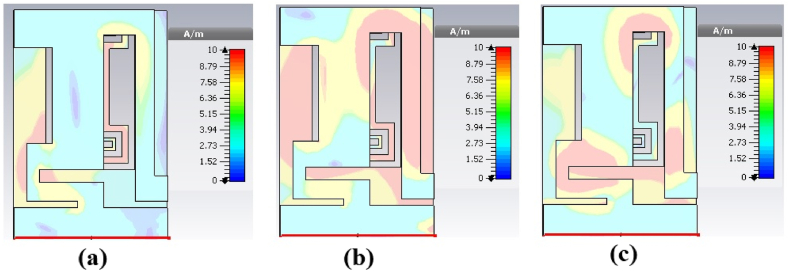
Surface currents of the proposed patch antenna at (a).2.45 GHz. (b). 5.8 GHz. (c). 8.8 GHz.

Fig. 9(a), 9(b) and 9(c) represent the current density distributions at the designed resonant frequencies. It is evident that most of the surface current is concentrated along the length of the meta-atom at 2.45 GHz and an insignificant magnitude of the current is directed towards the meander patch. At 5.8 GHz, significant magnitude of current density is concentrated along the length of the resonator and a portion of current is directed towards the ground plane. At 8.8 GHz, the maximum current is concentrated on the patch. An inconsiderable magnitude of current density is diverted towards the resonator and the ground plane.

**Fig. 10:**
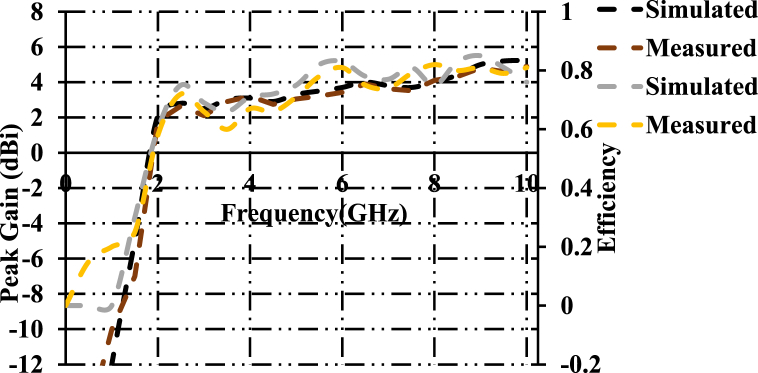
Peak gain and efficiency of the designed antenna structure.

Fig. 10 shows the measured and simulated curves of peak gain and efficiency. Simulated peak gains of 2.84dBi, 3.6dBc, and 4.4dBi are observed at the proposed resonant frequencies. Measured peak gains of 2.79dBi, 3.52dBc, and 4.36dBi are acquired at the computed frequencies. The simulated and measured efficiencies of 78%, 83.5%, and 82% and 77.6%, 82.3% and 84.1% are acquired.

**Fig. 11:**
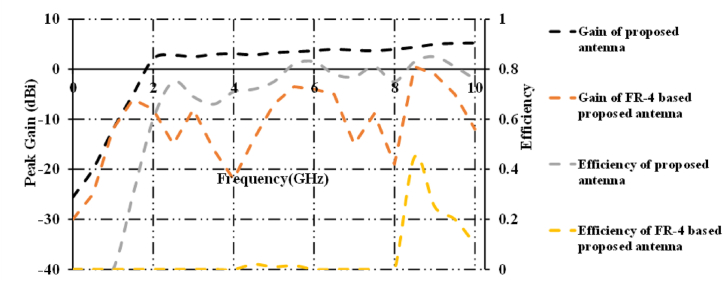
Comparision of gains and efficiencies of proposed antenna and FR-4 based patch antenna.

Fig. 11 shows gains and efficiencies of proposed patch antenna and FR-4 based patch antenna. It is clearly evident that the proposed patch antenna is superior than that of FR-4 based proposed patch antenna in terms of gains and efficiencies. A peak gain of 0.5dBi is observed at 8.45 GHz. An efficiency of 46% is obtained at 8.45 GHz.

**Fig. 12:**
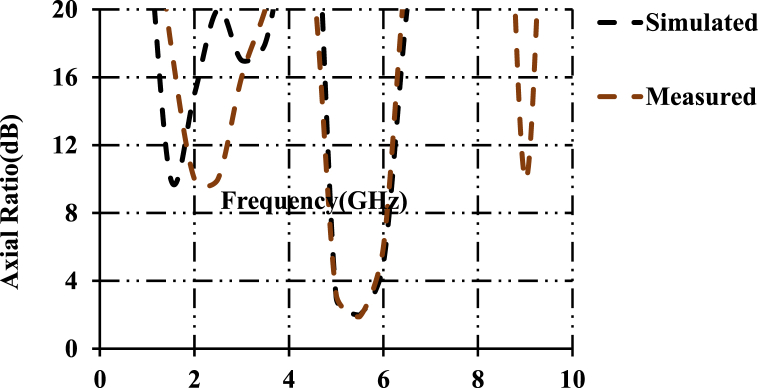
Axial ratios of the designed antenna.

**Fig. 13:**
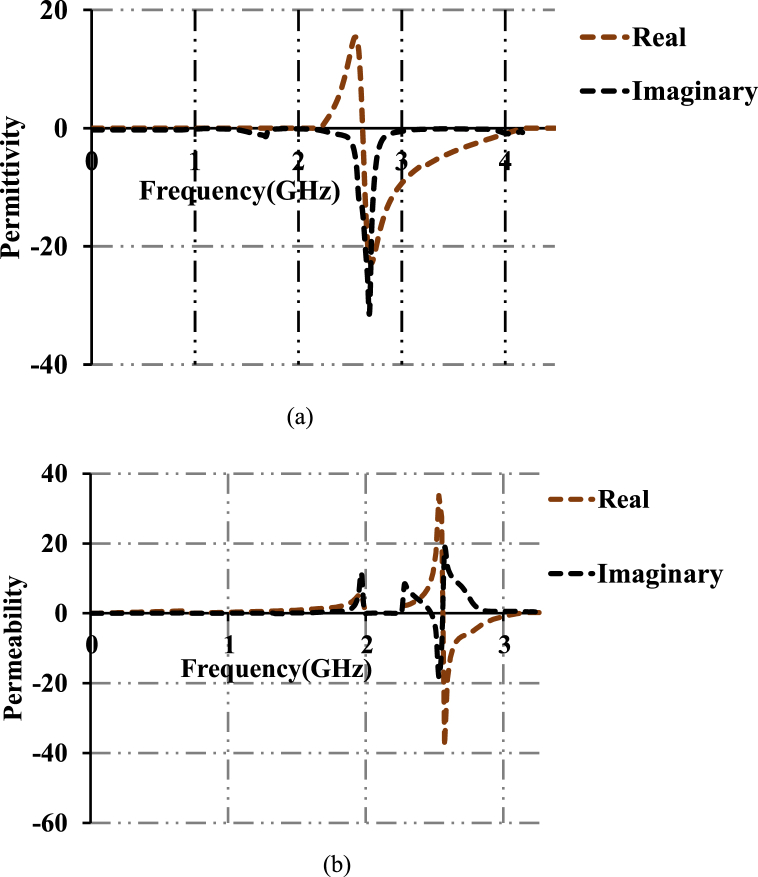
(a). Permittivity. (b). Permeability.

The simulated and measured axial ratios of the designed structure are shown in Fig. 12. Axial ratios of 21.34 dB, 2.1 dB and 20.2 dB are observed at computed resonant frequencies. The proposed patch antenna possesses linear polarization at first and third frequencies (2.45 GHz and 8.8 GHz). In the frequency range of 5.78GGHz∼5.81 GHz, the antenna possesses Right Hand Circular Polarization (RHCP). Fig. 13(a) and (b) show the permittivity and permeability plots of the proposed dumbbell meta-atom. Permittivity and permeability are calculated using the NSW (Nicolson-Ross-Weir method). This is performed by obtaining and computing S-parameters and further plots are plotted in MATLAB. The permittivity and permeability are going negative at 2.49 GHz. It can be concluded from the plots that the dumbbell-shaped meta-atom is a Double Negative Metamaterial (DNG MTM).

**Fig. 14:**
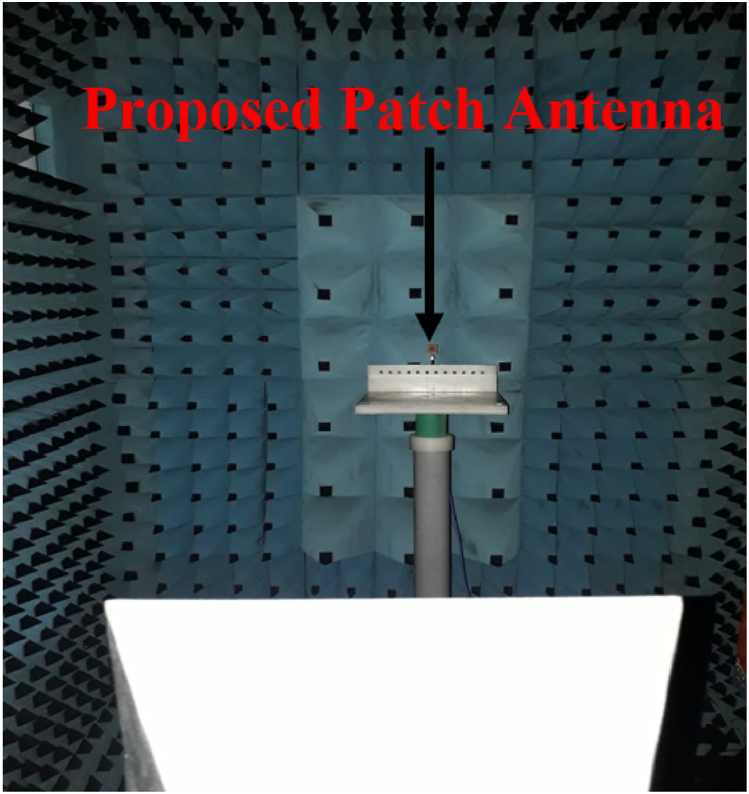
The antenna measurement setup.

**Fig. 15:**
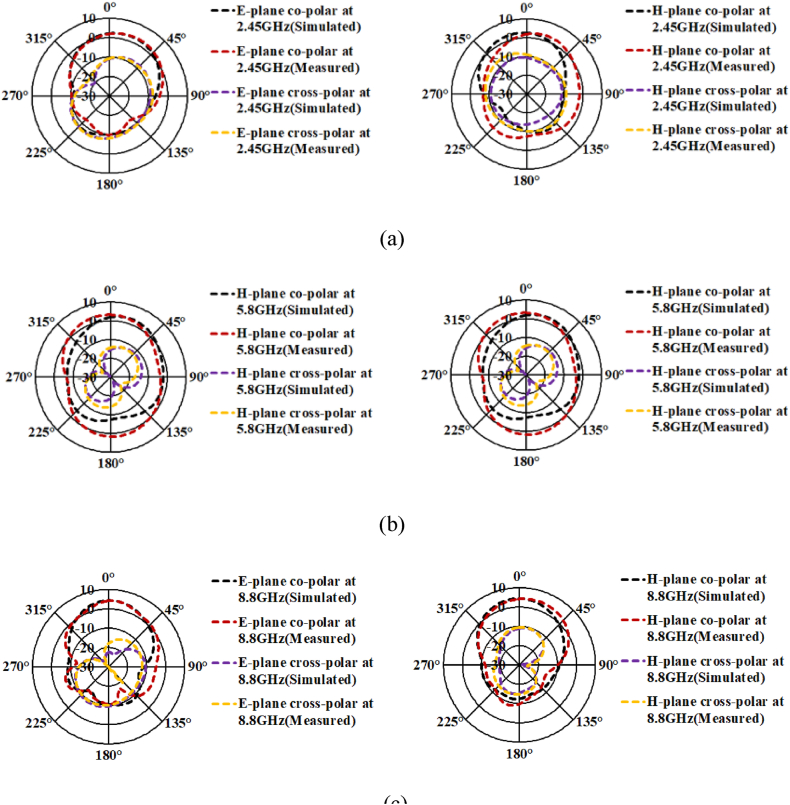
Radiation patterns of the proposed patch antenna at (a). 2.45 GHz. (b). 5.8 GHz. (c). 8.8 GHz.

Fig. 14 shows the radiation pattern measurement setup. The antenna patterns corresponding to all the radiating planes are simulated in CST-Microwave Studio and measurement is accomplished using double-ridge horn as the testing antenna. Fig. 15(a), 15(b), and 15(c) represent the radiation patterns in E− and H-planes at the resonant frequencies of interest. The measured radiation patterns at 2.45 GHz are unidirectional with the cross-polarization levels below −10dB as shown in Fig. 15(a). Measured and simulated gains of 2.72dBi and 2.75dBi are observed at 2.45 GHz. The antenna patterns at 5.8 GHz are unidirectional with the cross-polarization levels below −12dB as shown in Fig. 15(b). Fig. 15(c) Shows the measured and simulated gains of 3.45dBc and 3.5dBc at 5.8 GHz. The measured radiation patterns at 8.8 GHz are unidirectional with the cross-polarization levels below −13dB. Measured and simulated gains of 4.32dBi and 4.34dBi are observed at 5.8 GHz. Both simulated and measured results are in proximity and the difference in the results is due to human and measurement equipment errors.

Table 1 presents the detailed contrast between the present design and the preceding designs. The present design is weighed up with the antennas in the existing literature working in the frequency range 2.4 GHz–3.5 GHz in the lower side and 5 GHz–9 GHz in the upper side. This possesses improved performance parameters compared to that of existing antennas. The overall area of the proposed antenna structure is 180 mm^2^. The proposed patch antenna is superior in terms of compactness, and performance parameters.

**Table 1:** Comparison between existing antennas and the proposed patch antenna.

Ref	Dimensions (mm^2^)	Substrate	Type	Frequency(ies) (GHz)	Peak Gain(s) (dBi)	Efficiency(ies) (%)
[8]	1254.6	FR-4	Dual-band	2.41 & 2.7	4.14 & 4.24	Not Mentioned
[9]	260	FR-4	Triple-band	2.5, 3.5 & 5.5	1.5 to 2.7	60 to 80
[12]	529	FR-4	Triple-band	2.4, 3.5 & 5.5	2.5, 2.65 & 2.72	Not Mentioned
[13]	1200	FR-4	Single-band	2.4	3.3	Not Mentioned
[14]	585	FR-4	Single-band	2.45	2.33	Not Mentioned
[15]	1120	FR-4	Triple-band	2.5, 3.5 & 5.5	0, 0.7 & 1.6	86, 87 & 92
[16]	480	FR-4	Dual-band	2.4 & 5.45	2.2 & 1.9	62.3 & 93.5
[19]	1190.4	FR-4	Triple-band	2.4, 3.5 & 5.5	2.25, 3.72 & 2.71	Not Mentioned
[21]	600	FR-4	Dual-band	2.6 & 3.41	1.5 & 2.6	96.2
[23]	750	FR-4	Triple-band	2.4, 3.5 & 4.9	3.6, 2.95 &3.96	Not Mentioned
[24]	66.2596	FR-4	Single-band	2.45	−8	Not Mentioned
[25]	350	FR-4	Dual-band	2.45 & 3.6	3.76, & 3.44	92.5 & 94.8
[26]	400	FR-4	Dual-band	2.45 & 5.2	3.8 & 2.9	Not Mentioned
[27]	4340.89	FR-4	Single-band	2.45	3.12	Not Mentioned
This work	180	RT/Duroid 5880	Triple-band	2.45, 5.8 & 8.8	2.7, 3.6 &4.4	78.1, 83.5 &82

## 7 Conclusion

In this paper, a dumbbell-shaped meta-atom loaded meander shaped patch antenna is presented. The protype is designed using a low loss RT/Duroid-5880 substrate to avoid radiation leakages due to its compact size. The active radiating area is significantly reduced by 93% by introducing meta-atom. It has a total volume of 274.32 mm^3^ and exhibits Right Hand Circular Polarization (RHCP). The proposed patch antenna resonates at 2.45 GHz, 5.8 GHz, and 8.8 GHz. The structure exhibits impedance bandwidths of 110 MHz, 730 MHz, and 1.83 MHz in the frequency bands of (2.4 GHz–2.51 GHz), (5.13 GHz–5.86 GHz), and (7.7 GHz–9.53 GHz) respectively. Peak gains of 2.84dBi, 3.6dBc, and 4.4dBi are achieved desired resonant frequencies. The proposed antenna is an apt candidate for Wi-Fi/ISM/X-band applications. The proposed meta-atom loaded patch superior compared to the antennas proposed in the literature. The proposed antenna is limited to tri-band applications with reasonable gain and efficiency. The proposed antenna can be made quad band by introducing meta-surfaces which further improve the performance of the proposed antenna structure.

## Funding

This study was financially supported by 10.13039/501100002456Chonnam National University (Grant number: 2024-0926).

## Data availability statement

All the data are included in this paper is not deposited in any publicly available repository and will be made available on request.

## CRediT authorship contribution statement

**Suresh Angadi:** Conceptualization. **Karteek Viswanadha:** Data curation. **Ravikumar Chinthaginjala:** Funding acquisition, Formal analysis. **Dhanamjayulu C:** Investigation. **Kim Tai-hoon:** Project administration, Funding acquisition. **Kumar S:** Visualization.

## Declaration of competing interest

The authors declare the following financial interests/personal relationships which may be considered as potential competing interests: Tai-hoon Kim reports financial support was provided by 10.13039/501100002456Chonnam National University - Yeosu Campus. Tai-hoon Kim reports a relationship with Chonnam National University - Yeosu Campus that includes: employment. If there are other authors, they declare that they have no known competing financial interests or personal relationships that could have appeared to influence the work reported in this paper.
